# Heart and Lung Transplantation

**Published:** 1988-11

**Authors:** J. Wallwork

**Affiliations:** Papworth Hospital, Cambridge


### Heart and Lung Transplantation – Present and Future

**Mr J. Wallwork**
Papworth Hospital, Cambridge

Mr Wallwork began by emphasising that movement in this subject is so rapid that he could speak only of the situation today, tomorrow it will certainly have changed.

In 1979, when heart transplants started at Papworth there were only 5 centres active in the world, in 1987 there were 100 centres in the USA, probably over 55 centres in Europe. The heart-lung transplant service at Papworth started in 1984, and next year in 1988 it is planned to do 35 heart lung transplants.

The history begins with a Russian, Demikof who did an animal heart-lung transplant in 1946 and it survived two hours. Denton Cooley had a patient last 14 hours, Lillehei in 1969 had a patient with a few days survival, Christian Barnard did a heart transplant in 1971 that lasted 23 days. This was ‘it’ until the 1980s. About 59 single lung transplants were performed all ending in disaster.

Sir Roy Calne first used cyclosporin A as an immunosuppressive agent in 1979 in Cambridge. It was then used in heart transplantation in Stanford with success. In 1981 the first successful heart-lung transplant was done at Stanford and Mr Wallwork 'happened to be there'! In 1984 the first successful case in this country was done at Papworth and then in 1985 they started to take the organs from the donor at a distance which was a very important development.

The advantages of a heart along with the lungs are several, it replaces all the diseased tissue, it is technically simpler and the trachea heals better because it gets its blood supply from the coronary arteries.

Among diseases suitable for heart-lung transplantation are a whole group of congenital heart conditions with pulmonary hypertension, particularly children who have had difficult corrective or semi-corrective procedures in infancy and for whom there is no further possibility of anatomical correction, a few patients with primary pulmonary hypertension, emphysema, interstitial pulmonary disease and the cystic fibrotics who are becoming a very important group.

Selection of cases for this restricted service requires some harsh choices. Nobody over the age of 50 has yet been taken, surgery for the younger age group is increasing with collaboration with Great Ormond Street. Total referrals so far have been 300, many were unsuitable because they were too old or too ill. Emergency transplantation would not be the best use of our scarce resource. The waiting list contains more women than men in contrast to heart transplantation where only one in ten are female. 17 have died waiting. 33 heart-lung transplants have been done with a one year survival of about 78%. All patients were chronically disabled and many about to die from their disease. This is about the same survival rate as from heart transplantation. The waiting time for an appointment is about six months and a further 10 months on average before transplantation, varying from four weeks to two years. Those patients with pulmonary hypertension who are very ill have been given long term self-administered infusions of prostacycline, initially it was hoped it might reverse their pulmonary hypertension, it does not do this but they are dramatically improved and it buys them extra time. It is very expensive and is not used as a primary form of therapy.

The methods of handling donors have been developing at Papworth in quite a unique way and have been adopted by other heart-lung transplant centres. Lungs must be in good condition and able to work right away, unlike transplanted kidneys which can be supported initially by dialysis. Donors over 40 years old are not used. A major cause of recipient death in earlier series has been cytomegalovirus (CMV) infection. So all donors are tested for CMV and CMV positive organs are not put into CMV negative patients. Another important point is to get the size right – it is no good putting large organs into small patients and the size can be gauged from the X-rays and the height and weight of the donor.

Organs at the donor's hospital and the method of preserving them is very important. Organs must be cold and the lungs inflated. Lungs are perfused with a single cold flush. The solution is important, essentially blood from the donor with an extracellular solution of mannitol is used. The patient is given a prostacycline infusion to dilate all the vascular bed and put it in at 4 degrees under gravity. There have been no primary organ failures in 33 transplants. The ischaemic time, from removal to insertion, has been up to four hours. In some centres organs have been transported by aereoplane with complicated apparatus to perfuse and oxygenate them, but this is unneccessary and liable to fail.

An essential in the operative technique is to get the timing right. The operation to remove the recipients heart and lungs is begun at the same time as the operation at the donors hospital to remove the organs. The operation to remove the recipient's organs is the hardest part. The phrenic, the vagus and the recurrent laryngeal nerves must not be damaged. The whole trachea must be saved to preserve the cough reflex. It is easier to avoid damaging these important structures if the heart and the lungs are removed separately and not all together as was done originally. The operation becomes much more difficult if patients have adhesions from previous lung surgery. This can cause much bleeding and may rule them out as transplant recipients. Putting the organs in is much easier and takes only about 35 minutes – first join up the trachea, then the aorta and then the right atrium.

There is nothing special in postoperative management, patients are extubated usually in 48 hours, sometimes in 12 hours. Fluids are restricted to prevent the lungs from getting wet, they have lost their lymphatic connections and so will take a long time to get rid of oedema. The patients are got up as early as possible, and usually 3 or 4 days after surgery they are sitting in a chair exercising and beginning to become mobile.

The routine for prevention of rejection is basically with Cyclosporin A and Immuran. Steroids are given only for rejection episodes. The major problem is how to detect rejection in the lungs. The difficulty is to distinguish rejection from infection. The stethoscope, to detect new wheezes and crackles, simple pulmonary function tests – e.g. the FEV 1, and transbronchial biopsies may give evidence before there are X-ray changes. Rejection episodes generally occur within the first 3 months but these methods have picked up some later episodes. If missed they may go on to obliterative bronchiolitis and long term damage. Various infections may occur and all patients are given septrin when receiving steroids to prevent pneumocystus. They are barrier nursed for 3 days, they go into the general wards for 2 or 3 weeks and then out into the hospital flats for another week before going home.

Results – of the first 31 patients, age 12 to 46 and mostly women, 23 are alive and well. One is back at work at his job in the army, another plays very good squash. Their quality of life is good, this is especially important when the length of life is as yet unknown. Actuarial survival is 78% at one year and 68% at 2 years.

The full cost for the first year is about £15,000 per patient, or one tenth the cost of keeping a patient on prostacycline for a year.

In reply to a comment by Mr Keen, Mr Wallwork said that the surgery is now the straightforward part of the exercise and the organisation has been worked out. The long term results are going to depend on the physicians and the immumologists.

